# Understanding Attitudes Toward Zoster Vaccination in the Hospital Setting: A Multidisciplinary Model to Contrast Vaccine Hesitancy in Fragile Patients—A Prospective Longitudinal Observational Study

**DOI:** 10.3390/vaccines13080843

**Published:** 2025-08-08

**Authors:** Luca Regazzi, Silvia Martinelli, Federica Rizzo, Enrica Tamburrini, Pierluigi Francesco Salvo, Silvia Laura Bosello, Francesco Landi, Simona Sica, Antonietta Spadea, Domenico Pascucci, Patrizia Laurenti

**Affiliations:** 1 Hygiene Unit, Department of Life Sciences and Public Health, Università Cattolica del Sacro Cuore, 00168 Roma, Italy; 2 Department of Biomedicine and Prevention, Tor Vergata University of Rome, 00133 Roma, Italy; 3 Infectious Diseases Unit, Department of Safety and Bioethics, Università Cattolica del Sacro Cuore, 00168 Roma, Italy; 4 Infection Disease Unit, Department of Medical and Surgery Sciences, Fondazione Policlinico Universitario A. Gemelli IRCCS, 00168 Roma, Italy; 5 Rheumatology Unit, Department of Geriatric and Orthopaedic Sciences, Università Cattolica del Sacro Cuore, 00168 Roma, Italy; 6 Rheumatology Unit, Department of Ageing, Neurosciences, Head-Neck and Orthopaedics Sciences, Fondazione Policlinico Universitario A. Gemelli IRCCS, 00168 Roma, Italy; 7 Geriatrics Unit, Department of Geriatric and Orthopaedic Sciences, Università Cattolica del Sacro Cuore, 00168 Roma, Italy; 8 Geriatrics Unit, Department of Ageing, Neurosciences, Head-Neck and Orthopaedics Sciences, Fondazione Policlinico Universitario A. Gemelli IRCCS, 00168 Roma, Italy; 9 Hematology Unit, Department of Radiological and Haematological Sciences, Università Cattolica del Sacro Cuore, 00168 Roma, Italy; 10 Haematology Unit, Department of Laboratory and Hematology Sciences, Fondazione Policlinico Universitario A. Gemelli IRCCS, 00168 Roma, Italy; 11 ASL Roma 1—Local Health Authority, 00193 Rome, Italy; 12 Health Management, Fondazione Policlinico Universitario A. Gemelli IRCCS, 00168 Roma, Italy; 13 Department of Woman and Child Health and Public Health, Fondazione Policlinico Universitario A. Gemelli IRCCS, 00168 Roma, Italy

**Keywords:** vaccine hesitancy, herpes zoster, HZV, recombinant zoster vaccine, immunocompromised, fragile patients

## Abstract

**Background**: Immunocompromised and clinically fragile individuals are at increased risk of herpes zoster (HZ), but vaccine uptake remains low due to organizational barriers and vaccine hesitancy (VH). This study aimed to evaluate the impact of a multidisciplinary hospital-based counseling model on attitudes toward the recombinant adjuvanted zoster vaccine (RZV). The primary objective was to assess changes in VH over time using the Vaccination Attitudes Examination (VAX) scale; secondary objectives included identifying factors associated with VH, evaluating vaccine safety, and monitoring post-vaccination HZ incidence. **Methods**: A prospective cohort study was conducted in a large research hospital in Rome, in collaboration with the Local Health Authority. Eligible patients were offered individualized counseling and administered two doses of the RZV. VH was assessed using the 12-item VAX scale at baseline and at one-year follow-up. Multivariable linear regression analysis was performed to investigate associations between baseline characteristics and VH scores. **Results**: Between July 2022 and July 2023, 178 patients were enrolled, of whom 90 completed the one-year follow-up. Baseline VH was moderate (mean VAX: 2.291/6.000); higher scores were significantly associated with younger age, female sex, and rheumatologic disease (*p* < 0.05). After the intervention, VAX scores improved significantly across all subscales, particularly in trust in vaccine safety and benefits (*p* < 0.001). RZV was well tolerated; adverse events were mild and transient. Breakthrough HZ occurred in 3.33% of cases during follow-up. **Conclusions**: A multidisciplinary hospital-based model effectively improved vaccine attitudes and acceptance in fragile patients. Tracking VH over time with validated tools offers insights for scaling targeted interventions in high-risk groups.

## 1. Introduction

Herpes zoster (HZ) occurs worldwide with no seasonal variation in incidence. The incidence of HZ is age-dependent and ranges from 1.2 to 3.4 per 1000 persons per year among young people to 3.9 to 11.8 per 1000 persons per year in elderly patients (i.e., >65 years) [1]. According to a systematic review of studies from 2002 to 2018, the cumulative incidence was estimated to be between 2.9 and 19.5 cases per 1000 population, with a predominance of women, specifically in individuals ≥ 50 years of age [2]. Common risk factors for HZ are age > 50 years, immunosuppression, infections, and mental stress [3]. According to the Global Burden of Disease database [4].

HZ vaccines aim to prevent HZ activation and the development of postherpetic neuropathy (PHN). Two HZ vaccines are currently available, a live attenuated vaccine (Zostavax; Merck, Kenilworth, NJ, USA) (VZV) and a recombinant vaccine adjuvanted with the glycoprotein E subunit of VZV (Shingrix, GlaxoSmithKline, London, UK) (RZV) [5].

A Chinese study analyzed the potential public health impact of vaccination with RZV, compared with the status quo of no vaccination, in individuals aged ≥50 years in Beijing. It estimated that mass vaccination with RZV could prevent >430,000 cases of HZ and >51,000 cases of PHN compared with no vaccination. The authors suggested that >14,000 hospitalizations and >1,000,000 outpatient visits could be prevented [6]. The efficacy of RZV against HZ has reached 85.5% [7], and it is the only vaccine that is safe even for immunocompromised people [8,9]. The reason for this could be that RZV has a technology based on glycoprotein E (gE), which is the most abundant surface glycoprotein of VZV and plays a key role in virus entry, making it a key target for both humoral and cellular immune responses. In addition, gE is essential for cell-to-cell spread, and antibodies against gE are easily detectable in individuals with previous exposure to VZV. The adjuvant system of RZV, AS01B, is critical for inducing robust and persistent antibody and CD4+ T cell responses, which are particularly important in immunocompromised individuals [5].

However, despite strong evidence supporting the efficacy of vaccines in preventing communicable diseases, vaccination coverage remains suboptimal, particularly among individuals who are vulnerable due to immunocompromise or immunosenescence [10]. Multiple and interrelated factors contribute to this issue. These include low vaccine recommendation rates by specialists (such as rheumatologists, hematologists, infectious disease physicians, and general practitioners), limited confidence in vaccine effectiveness, and the variable and often reduced immune response in frail patients [11,12]. Additionally, vaccine hesitancy (VH)—driven by safety concerns, lack of trust in physicians or health authorities, and broader skepticism towards pharmaceutical companies or government institutions—represents a major barrier to immunization efforts [13,14]. Structural and financial barriers may further reduce access to vaccines, especially in countries where costs are not fully covered by national health systems or insurance providers [15].

In Italy, recent studies have highlighted specific challenges to adult immunization. These include the absence of systematic monitoring of vaccine coverage for certain recommended adult vaccines—such as the herpes zoster vaccine—and significant disparities in service availability across regions [16]. Furthermore, as noted by Cesaroni et al. [17], social and economic inequalities—such as lower educational attainment and immigrant status—are associated with lower vaccine uptake in the Lazio region. Although official institutional data are lacking, available studies indicate that in Italy, overall HZ vaccination uptake among at-risk adults is very low (~9.6%), with significant regional variability; the northeast achieves up to 26.9%, while islands register under 3% [18]. In the Lazio region, specifically among fragile patients served by ASL Roma 2, coverage is even lower (~1.5%) [19].

In response to these challenges, both international and national health authorities have emphasized the importance of strengthening immunization strategies targeting at-risk populations. In Italy, the National Vaccine Prevention Plan (PNPV) 2023–2025 identifies the vaccination of adults with congenital or acquired immunodepression as a public health priority, recommending integrated and proactive approaches that reinforce existing vaccination services [20].

To overcome access and adherence barriers, health systems should adopt adaptive public health models, including the establishment of vaccination services within hospitals, especially for clinically fragile populations. Such models can foster a more personalized approach, improving patient engagement and trust, and promoting a shift from passive acceptance to active confidence in vaccination [21].

In the Lazio region of Italy, vulnerable adults with congenital and/or acquired immunodepression are currently vaccinated in outpatient vaccination clinics run by the out-of-hospital health services of the Local Health Authority. If we exclude vaccination for SARS-CoV2, in Italy, there are only a few vaccination settings in hospitals offered to frail patients.

At the time of the present study, the Lazio region included the recombinant zoster vaccine (RZV) in its immunization offer for adults aged ≥18 years presenting at least one of the following conditions: congenital and/or acquired immunodepression, history of recurrent herpes zoster, splenectomy, or dialysis. The vaccine was provided free of charge within this target population [22]. The recommended schedule consisted of two intramuscular doses administered 2 to 6 months apart, in accordance with the Summary of Product Characteristics [23]. It should be noted that, as of December 2024, the target population eligible for free RZV vaccination in Lazio has been further expanded [24].

Considering these challenges, the present study aimed to evaluate, within a hospital setting, the impact of a multidisciplinary vaccine counseling model on attitudes toward the RZV among immunocompromised or clinically fragile patients. Specifically, the primary objective was to assess changes in VH over time, as measured by the validated Vaccination Attitudes Examination (VAX) scale, before and after the counseling and vaccination intervention. Secondary objectives included the identification of demographic and clinical factors associated with VH, the assessment of vaccine safety through monitoring adverse events, and the evaluation of the incidence of post-vaccination herpes zoster infections.

## 2. Materials and Methods

### 2.1. Study Design

The study is a prospective longitudinal observational single-center cohort study, which had duration of 12 months (July 2022–July 2023) with a further 12 months of follow-up (until July 2024). The selected patients were all followed up at Fondazione Policlinico Universitario A. Gemelli (FPG), in the Hospital Units (HUs) of Infectious Diseases, Hematology, Rheumatology, Nephrology and Geriatrics. Although recruitment was primarily conducted within the core Hus, a limited number of patients from other HUs were included upon the initiative of physicians who were aware of the project and referred eligible patients meeting the inclusion criteria. FPG is a tertiary-level academic hospital that serves as a referral center for complex cases and provides care to a population of approximately 1.5 million people across the Lazio region [25]. The selection of patients had focused primarily on a convenience sample of high-risk individuals (e.g., active chemotherapy, severe forms of HZ, etc.) for which vaccination is crucial in order to prevent serious complications.

The inclusion criteria, in accordance with the directions of the Lazio region [22], were confirmed as the diagnosis of congenital and/or acquired immunodepression and/or previous relapsed HZ and/or splenectomy/dialysis and age > 18 years, while the exclusion criteria were pregnant and lactating women, or those who had reported episodes of hypersensitivity to the active ingredients and excipients contained in the vaccine formulation.

During the project, demographic data (age, sex), clinical data (pathological and pharmacological history, risk factors for serious and/or invasive infectious diseases such as transplantation, splenectomy, HIV, oncological diseases) and vaccination practice (past infections, vaccines and adverse effects) were collected from each patient’s medical record and entered a database. Each patient signed a written informed consent before vaccination and data collection.

Several strategies were implemented a priori to reduce potential sources of bias (Supplementary Methods S1).

The study was submitted to the ethics committee of FPG (Prot. No. 0020857/22 dated 06/17/2022 SECRETARY ETHICS COMMITTEE; Protocol Code: Ce VOT-Her Zo; Prot. ID No. 5001 STUDY NO PROFIT), conducted in full compliance with the latest revision of the Declaration of Helsinki and according to the Good Clinical Practice guidelines. Participation in the study was entirely voluntary, with individuals joining upon providing written informed consent. Stringent measures were implemented to ensure the security and confidentiality of participants’ data.

### 2.2. Endpoints of the Study

The primary endpoint of the study was to assess the level of VH in the hospital setting as measured by the Vaccination Attitudes Examination (VAX) scale, both overall and stratified by demographic and clinical variables.

The secondary endpoints were to assess changes in vaccine hesitancy of a subsample of patients adhering to the vaccine proposal by comparing the pre-counseling VAX scale score with the post-counseling score, and to assess the possible presentation of adverse reactions to vaccination or HZ breakthrough infection after vaccination.

### 2.3. Timing of the Study

The study was structured into three main phases: [T1] baseline assessment including administration of the VAX scale; [T2] multidisciplinary vaccination counseling and administration of the recombinant zoster vaccine (RZV); and [T3] a one-year follow-up with reassessment of vaccine hesitancy and evaluation of adverse events or breakthrough infections. A detailed description of each phase is provided in Supplementary Methods S1.

### 2.4. Description of the Assessment Tool

Attitudes and intentions toward vaccines were assessed using the Italian adaptation of the 12-item Vaccination Attitudes Examination (VAX) scale [26], which captures key dimensions of vaccine hesitancy. A detailed description of the instrument, scoring procedures, and its psychometric properties is available in Supplementary Methods S1.

### 2.5. Statistical Analysis

The analysis was conducted in two main stages. In the first stage, the full patient sample was analyzed to identify the determinants of vaccine hesitancy. In the second stage, a subsample of patients was examined to assess the potential impact of the intervention on attitudes toward vaccination.

The internal consistency of the VAX Scale was assessed using both Cronbach’s alpha and McDonald’s Omega to ensure reliability for both the full sample and the recalled subsample, before and after the intervention. Descriptive statistics were computed for both the full sample and the recalled subsample. Continuous variables were summarized as means with standard deviations (SD), medians, and interquartile ranges (IQR). Categorical variables were presented using frequency counts and percentages.

To explore the determinants of vaccine hesitancy, cross-tabulations and multiple linear regression models were employed. The VAX scale total score and each of its four subscale scores (Mistrust of Vaccine Benefit, Worries about Unforeseen Future Effects, Concerns about Commercial Profiteering, Preference for Natural Immunity) were used as dependent variables. Independent variables included gender, age, and medical department.

Comparative analyses were conducted between participants who responded to the recall survey and those who did not respond to evaluate the representativeness of the subsample and identify potential attrition bias. Two-sample *t*-tests were performed to assess differences in continuous variables, while chi-square tests were used to compare categorical variables between the two groups. The distribution of the differences between continuous variables before and after the intervention was assessed using Q-Q plots, histograms, and the Shapiro–Wilk test for normality. As all differences appeared to violate the normality assumption, permutation-based paired *t*-tests with Welch’s correction were utilized to compare continuous variables before and after the intervention.

In the recalled patient subsample, adverse effects and breakthrough infections were analyzed on a case-by-case basis and described qualitatively rather than quantitatively. Given the minimal missing data (less than 2% across key variables), a complete case analysis was performed to handle missing values.

All statistical analyses were conducted using Stata 17 and R software (version 4.2.2, CRAN^®^) within the RStudio platform (version 2024.04.1+748, © 2009–2022 RStudio, PBC). Statistical significance was set at *p* < 0.05 for all analyses.

## 3. Results

From July 2022 to July 2023, 178 patients underwent vaccine counseling receiving two doses of RZV and 90 of them were recalled after 365 days from the second dose (Table 1).

Considering all patients, 87.64% of participants were aged 45 or older. The largest age group was the over-65 age group (47.19%), followed by the 45–64 age group (40.45%), while only 12.36% of participants fell in the 18–44 age group (Table 1). The gender distribution, on the contrary, was quite balanced, with a slight female majority (51.69% women vs. 48.31% men) (Table 1). The primary diseases from which patients were affected were HIV infection (34.27%), herpes zoster (HZ) relapses or severe forms (23.03%), and rheumatologic diseases under immunosuppressive therapy (21.35%), with a significant majority (67.98%) reporting at least one pre-existing concomitant clinical condition (Table 1).

Comparing the group of recalled patients to the group of patients who were lost to follow-up, none of the categorical variables (age groups, gender, previous zoster, primary indication and hospital department) showed statistically significant differences (Table 1). Similarly, most continuous variables, including age, mistrust of vaccine benefit, worries about unforeseen future effects, concerns about commercial profiteering, and vaccination interval, showed no statistically significant differences between the two groups (Table 1). However, three variables showed significant differences: vaccination interval (*p* = 0.0319), preference for natural immunity (*p* = 0.00123), overall vaccine hesitation score (*p* = 0.0311) (Table 1). Cronbach’s alpha and McDonald’s omega of VAX scale for the whole sample of 178 patients were, respectively, 0.9107 and 0.9105, which indicates an excellent internal consistency (i.e., the items in the scale measured the same underlying construct (vaccine hesitancy) consistently). The same was confirmed for each of the subcategories.

The mean VAX score for the whole sample was 2.291 (SD: 0.936) on a 6-point scale, indicating a moderate level of vaccine hesitation overall (Table 1). Analyzing the subscale results, “concern about unanticipated future effects” scored the highest, while “distrust in vaccine benefits” scored the lowest (Table 1).

The interval between the two doses administered was 64 days (IQR: 16 days) with a range of 21 to 204 days (Table 1). The relationship between the mean scores of the VAX scale/subscales and the characteristics of participants is shown in Table 2. Younger participants (0–44) showed greater hesitation toward the vaccine (2.53 ± 1.08), with a clear tendency to decrease with increasing age (2.08 ± 0.8 for 65+ patients), while rheumatology patients showed the greatest hesitation both overall (score: 2.54 ± 1.04) and for all specific subdimensions (Table 2).

**Figure 1:**
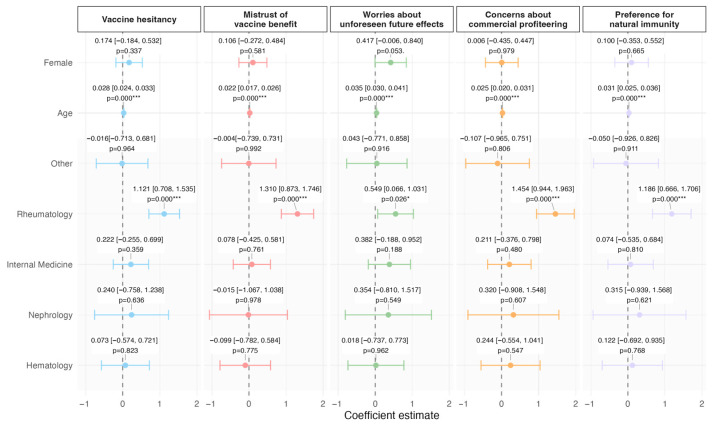
Regression coefficients for factors associated with vaccine hesitancy and with its subdimensions, as measured by the VAX Scale. This forest plot displays the estimated regression coefficients (points) and their 95% confidence intervals (horizontal bars) for various demographic and clinical predictors. The panels represent the different dimensions of vaccine hesitancy: overall vaccine hesitancy (first column), followed by its four sub-dimensions derived from the VAX Scale (mistrust of vaccine benefit, worries about unforeseen future effects, concerns about commercial profiteering, and preference for natural immunity). The x-axis, representing the coefficient estimate, is common across all panels for direct comparison. *p*-values are presented alongside each estimate, with significance levels defined as follows: *** *p* < 0.001, * *p* < 0.05.

Regression analysis results show that age was strongly associated with vaccine hesitancy overall (*p* < 0.01, Figure 1, Table S1), as well as with all four subdimensions of the VAX scale (*p* < 0.01, Figure 1, Supplementary Tables S2–S5) Similarly, being a patient of the Rheumatology Hospital Unit was associated with vaccine hesitancy overall (*p* < 0.01, Figure 1, Table S1), as well as with all four subdimensions of the VAX scale (*p* < 0.05 for worry about unforeseen side effects, *p* < 0.01 for the other three subdimensions, Figure 1, Tables S2–S5). Finally, being female was associated with higher worries about unforeseen future effects when compared to males below statistical significance (*p* < 0.1, Figure 1, Table S3).

**Figure 2:**
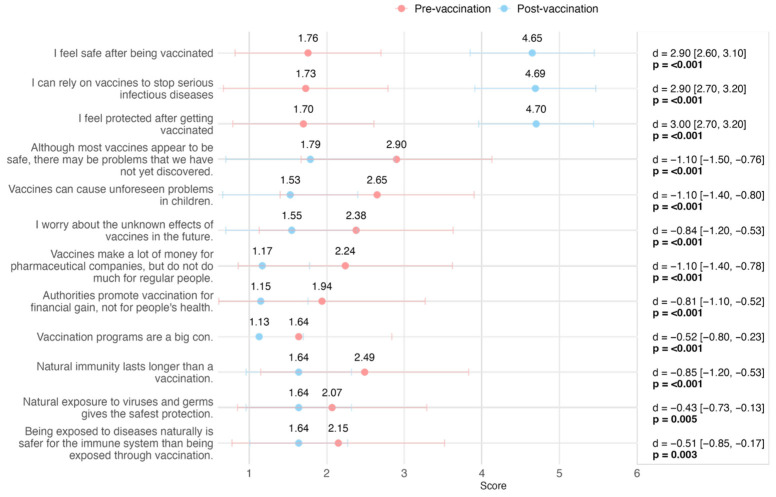
Pre- and post-vaccination mean scores on VAX scale items. Mean scores ± standard deviation are shown for each item, categorized by construct. Differences (Δ), 95% confidence intervals (CI), and *p*-values compare pre- and post-vaccination responses.

Comparison of responses to individual items on the VAX scale in the first phase of the project [T1] and in the patient recall phase [T3] showed significant changes across all items (Figure 2, Table 3). Specifically for positively framed statements about vaccines (e.g., “I feel safe after being vaccinated”), there were large positive changes, with differences ranging from 2.9 to 3.0 points. On the other hand, for statements or misconceptions about vaccines (e.g., “Vaccines can cause unexpected problems in children”), there were negative changes, indicating a reduction in agreement with these statements after the intervention. The greatest positive changes were recorded in statements related to feeling safe and secure after vaccination (+2.9, +3) and relying on vaccines to stop serious infectious diseases (+2.9). The largest reductions in agreement occurred with statements suggesting that vaccines are promoted for financial gains rather than health benefits (down to −1.10) and that vaccines can cause unforeseen problems (down to −1.10). Reductions in agreement were smaller, but still significant, with statements favoring natural immunity over vaccination (ranging from −0.51 to −0.85) (Figure 2, Table 3).

Finally, regarding side effects after the first and second doses, most participants did not report them (first dose: 80% without effects; second dose: 74.44% without effects), and those reported mostly represent minor side effects such as fatigue, chills, and fever (Table 4). The incidence of herpes zoster cases after vaccination (breakthrough infections) was 3.33% of participants (three cases): one case of oral herpes zoster, one case of both oral and ophthalmic herpes zoster, and one case of lumbar herpes zoster (Table 4).

## 4. Discussion

The aim of this study was to evaluate the level of VH among immunocompromised patients receiving the recombinant zoster vaccine RZV and to assess the impact of multidisciplinary vaccine counseling on reducing hesitancy and improving adherence. Additionally, the study sought to explore the safety and occurrence of breakthrough infections following vaccination in this high-risk population.

Despite the initial presence of moderate hesitancy, with a mean VH score of 2.291 (SD: 0.936), the data indicate that targeted interventions can positively influence patients’ attitudes towards vaccination. Notably, the proposed vaccination adherence rate reached 100%, significantly surpassing the vaccine adherence rates typically reported globally for RZV candidates [27,28]. The findings show a moderate level of vaccine hesitancy in the studied population, with younger patients (18–44 years) demonstrating higher levels of hesitancy compared to older groups, particularly those over 65.

Vaccine hesitancy is especially prevalent among younger individuals, both in the general population and within high-risk groups like those with HIV or other immunocompromising conditions (Table S1). This hesitancy often stems from concerns about vaccine safety, long-term side effects, and distrust of health messaging [29,30,31]. Immunocompromised individuals, despite being at a higher risk for severe illness, tend to show lower vaccine uptake, partly due to confusion regarding vaccine efficacy and fears of adverse effects [32]. The observations above are further confirmed by the analysis of the subscales “concern about unanticipated future effects” and “distrust in vaccine benefits,” which, like in this study, were measured using the VAX scale and are consistent with the literature that employed the same tool to assess vaccine hesitancy.

As discussed by Cummings et al. [33], vaccine hesitancy often stems from fears that vaccines, especially those developed rapidly, may have unknown long-term effects. This mirrors the concerns expressed by participants in our study, suggesting that uncertainty around vaccine safety remains a significant barrier. Additionally, Huynh [34] highlights the role of misinformation in fostering distrust in vaccine efficacy, a trend also evident in our findings. The skepticism observed is frequently fueled by narratives that downplay vaccine effectiveness, underscoring the importance of targeted educational efforts to mitigate these fears and rebuild public confidence.

Statistical significance (*p* values < 0.001 for most items) suggests that the changes observed in VAX scale responses between the first phase (T1) and the recall phase (T3) of our study are unlikely to be due to chance (Table 3). These results indicate a substantial positive shift in attitudes toward vaccines following the intervention, with an increase in confidence in their safety and efficacy, and a reduction in agreement with common misconceptions. These positive changes are largely attributable to the innovative multidisciplinary vaccination center model we adopted, which focuses on comprehensive, patient-centered care.

The significant positive changes observed in responses to positively framed statements such as “I feel safe after being vaccinated” (+2.9 to +3.0 points) and “vaccines stop serious infectious diseases” (+2.9) align with findings from other studies that have adopted innovative multidisciplinary models for vaccinations. Ibenyenwa et al. [35] and Shapiro Ben David et al. [36] emphasize that these models, which combine personalized counseling with a broad range of expertise, effectively address patient concerns, particularly around vaccine safety and long-term effects.

Similarly, the reduction in agreement with misconceptions like “vaccines can cause unforeseen problems” (down to −1.10) and “vaccines are promoted for financial gains” (down to −1.10) mirrors findings from Moosa et al. [37] and Baumann et al. [38], where such multidisciplinary approaches helped dispel misinformation, particularly in high-risk groups. Smaller but significant reductions in favor of natural immunity (−0.51 to −0.85) further reflect the positive impact of continuous counseling, as noted by Chau et al. [39].

Regarding safety, most participants did not report major side effects following vaccination. Minor effects, such as fatigue and localized reactions at the injection site, were the most commonly noted. Breakthrough infections of herpes zoster were rare, with 3.33% of participants experiencing mild cases after the second dose. This rate is slightly higher than the 2.94% reported in the study by Zerbo et al. [40], which included a larger sample and a longer follow-up period. The difference may be explained by the fact that our study involved high-risk patients with underlying clinical conditions, potentially accounting for the slight increase in breakthrough infections.

At the macro level, this study provides strong evidence for integrating multidisciplinary vaccination approaches into national and regional health strategies. Policymakers should prioritize the development of programs that focus on high-risk populations, such as the frail and immunocompromised, ensuring comprehensive, patient-centered care that addresses individual needs and disease contexts. At the meso level, hospitals and local health authorities should adopt the multidisciplinary model by integrating vaccine counseling into routine care for vulnerable patients, as suggested by several vaccine studies [41,42]. To make this operational, organizations should reinforce the model with structured professional training, audit-and-feedback, and point-of-care decision support that enable clinicians to address hesitancy effectively. In line with Italian evidence that patient uptake is highly dependent on physician advice [43] and that coverage can remain low even when attitudes are positive [44], these system-level supports are essential to ensure consistent and proactive vaccine recommendation practices across services. At the micro level, healthcare professionals—including physicians, nurses, and allied health workers—should employ a tailored approach when discussing vaccines. By creating individualized vaccination schedules based on the patient’s disease status, particularly during quiescent phases and before initiating immunosuppressive therapies or chemotherapy, as recommended in the recent literature [45,46,47], professionals can address specific concerns. Regular patient follow-up should be integrated into routine care to reinforce trust and ensure high adherence in at-risk populations, as shown in this study’s results.

The aforementioned results should be interpreted in light of the assessments of the strengths and weaknesses of the study.

One potential weakness is that the sample size may not fully represent the broader frail or immunocompromised population, limiting the generalizability of the findings. Moreover, patients were selected using a non-probability sampling technique based on accessibility and proximity to the researcher, which could introduce selection bias. Additionally, no prospective sample size calculation was performed. While the observed statistical significance suggests sufficient power to detect the effects found, this does not guarantee that the study was adequately powered to detect clinically meaningful differences. Future studies should include formal sample size estimation based on effect sizes observed here.

The absence of a control group also constitutes a significant limitation, as it prevents direct comparisons between the multidisciplinary approach adopted in this study and standard vaccination protocols. Additionally, since no unvaccinated individuals were included, it was not possible to assess the overall effectiveness of the vaccine.

Finally, a further limitation of our study is the relatively high loss to follow-up, with only 51% of the original cohort included in the one-year analysis. This was due to the inability to reach many participants by phone despite repeated contact attempts. While no differential exclusion criteria were applied at this stage, the reduced follow-up rate introduces the possibility of attrition bias, as those lost to follow-up may differ systematically from those who were reassessed. Comparative analyses suggest that patients lost to follow-up had higher baseline vaccine hesitancy, raising the possibility that our one-year findings may underestimate hesitancy or overstate the intervention’s impact. Therefore, the long-term results should be interpreted with caution. Despite these limitations, the study presents several noteworthy strengths. First, although it was a pilot study, it achieved a 100% acceptance rate among those offered the vaccine. This high level of adherence was supported by the use of personalized vaccination schedules, tailored to each patient’s clinical condition, which optimized timing for both safety and compliance. Furthermore, the analyses were conducted with rigorous statistical and methodological precision, enhancing the reliability of the results.

Another major strength is the 365-day follow-up period, which provided valuable insights into the long-term safety profile of the vaccine and enabled the observation of breakthrough infections. Notably, the smaller group of patients recalled for follow-up remained statistically representative of the entire cohort, as no significant differences were observed between pre- and post-vaccination data.

However, the interval between the administration of the two vaccine doses and the subsequent patient recall—intended to monitor the onset of any side effects—may have introduced recall bias. Additionally, although the study population was predominantly composed of older and frail individuals, this reflects the specific focus on high-risk patients rather than the deliberate exclusion of younger participants.

Finally, the use of the validated Vaccination Attitudes Examination (VAX) scale to assess vaccine hesitancy demonstrated excellent internal consistency. This ensured accurate measurement of patient attitudes and provided a reliable basis for comparison with similar studies.

Further studies are necessary to improve the generalizability of the findings, as the single-center design focused on frail, high-risk patients and did not fully represent the broader population recommended for vaccination by the Lazio region. Moreover, although the VAX scale was used to measure hesitancy, factors like socioeconomic status, cultural influences, and healthcare access were not examined. In addition, the analysis did not include a stratification by immunosuppressive drug class or investigate possible associations between specific medications (e.g., biologics, JAK inhibitors) and vaccination uptake or hesitancy. Future studies should address these aspects to provide a more comprehensive understanding of the determinants of vaccine acceptance in immunocompromised individuals.

## 5. Conclusions

In conclusion, this study demonstrates that a multidisciplinary approach to vaccine counseling can significantly reduce vaccine hesitancy and improve adherence among frail, high-risk patients. The high acceptance rate, combined with personalized vaccination schedules, highlights the effectiveness of integrating patient-centered care into vaccination programs. These findings underscore the importance of further expanding such models to broader populations, ensuring the effective implementation of vaccination strategies in vulnerable groups.

## Figures and Tables

**Table 1:** Baseline characteristics and vaccine hesitancy scores of study participants (N = 178): recalled patients (N = 90) and patients lost to follow-up (N = 88).

	All Patients (N:178)			Recalled Patients (N:90)			Patients Lost to Follow-Up (N:88)			Recalled Patients vs. Patients Lost to Follow-Up
Characteristic	n. (%)	Mean ± SD	Median (IQR)	n. (%)	Mean ± SD	Median (IQR)	n. (%)	Mean ± SD	Median (IQR)	*p*-Value
**Demographic Characteristics**										
**Age (years)**		61.91 ± 15.39	63.5 (19)		61.16 ± 13.16	62 (16.75)		62.68 ± 17.41	66 (17)	0.5111 ^†^
**Age groups**										0.1529 ^‡^
18–44	22 (12.36%)			12 (13.33%)			7 (7.95%)			
45–64	72 (40.45%)			41 (45.56%)			31 (35.23%)			
65+	84 (47.19%)			37 (41.11%)			47 (53.41%)			
**Sex**										0.0711 ^‡^
Male	86 (48.31%)			50 (55.56%)			52 (59.09%)			
Female	92 (51.69%)			40 (44.44%)			36 (40.91%)			
**Clinical Characteristics**										
**Previous zoster**										0.5546 ^‡^
No	126 (70.79%)			66 (73.33%)			60 (68.18%)			
Yes	52 (29.21%)			24 (26.67%)			28 (31.82%)			
**Primary indication for vaccination**										0.3183 ^‡^
Acquired immunodeficiency syndrome	61 (34.27%)			35 (38.89%)			26 (29.55%)			
Rheumatological diseases under treatment	38 (21.35%)			18 (20.00%)			20 (22.73%)			
Subjects with recurrences or particular forms HZ	41 (23.03%)			19 (21.11%)			22 (25.00%)			
Oncohematologic diseases under treatment	14 (7.87%)			8 (8.89%)			6 (6.82%)			
Other indications	24 (13.48%)			10 (11.11%)			14 (15.90%)			
**Hospital department**										0.5182 ^‡^
Infectious Diseases	62 (43.36%)			38 (42.70%)			34 (38.64%)			
Rheumatology	26 (18.18%)			19 (21.11%)			26 (29.55%)			
Geriatrics Medicine	25 (17.48%)			14 (15.56%)			13 (14.77%)			
Nephrology	7 (3.93%)			2 (2.22%)			5 (5.68%)			
Hematology	13 (7.30%)			8 (8.89%)			5 (5.68%)			
Other departments	30 (20.98%)			9 (10%)			5 (5.68%)			
**Vaccine Hesitancy**										
Mistrust of vaccine benefit		1.85 ± 1.09	1.33 (1.17)		1.73 ± 0.88	1.50 (1.00)		2 ± 1.28	1.33 (1.33)	0.1258 ^†^
Worries about unforeseen future effects		2.77 ± 1.06	2.67 (1.67)		2.65 ± 1.07	2.67 (1.33)		2.91 ± 1.02	3 (1.34)	0.1017 ^†^
Concerns about commercial profiteering		2.09 ± 1.29	1.67 (1.67)		1.95 ± 1.17	1.67 (1.67)		2.26 ± 1.4	1.67 (2)	0.1215 ^†^
Preference for natural immunity		2.45 ± 1.18	2.33 (1.67)		2.24 ± 1.10	2.33 (1.67)		2.7 ± 1.22	2.67 (1.66)	0.0123 ^†^*
Overall hesitancy score		2.29 ± 0.94	2.08 (1.37)		2.14 ± 0.85	1.83 (1.41)		2.46 ± 1.01	2.17 (1.25)	0.0311 ^†^*
**Vaccination Interval**										
Days between doses		74.46 ± 42.58	64 (16)		67.62 ± 22.17	64 (13.75)		81.45 ± 55.57	64 (27.25)	0.0319 ^†^*

^†^ indicates *p*-value from *t*-test for means for continuous variables. ^‡^ indicates *p*-value from chi-square test for proportions for categorical variables. * indicates statistically significant difference (*p* < 0.01).

**Table 2:** VAX scale and subscale scores by demographic and clinical characteristics.

Summary Statistics: Sex	Hesitancy Tot *	Mistrust of Vaccine Benefit *	Worries About Unforeseen Future Effects *	Concerns About Commercial Profiteering *	Preference for Natural Immunity *
Female	2.39 (0.91)	1.93 (1.14)	2.93 (1.02)	2.18 (1.29)	2.51 (1.13)
Male	2.18 (0.95)	1.75 (1.01)	2.59 (1.06)	2 (1.28)	2.38 (1.23)
**Summary statistics: age class**					
18–44	2.53 (1.08)	2.10 (1.21)	2.96 (1.23)	2.20 (1.42)	2.87 (1.25)
45–64	2.44 (0.98)	2.09 (1.13)	2.86 (1.06)	2.28 (1.39)	2.55 (1.22)
65+	2.08 (0.80)	1.55 (0.94)	2.63 (0.99)	1.89 (1.12)	2.24 (1.08)
**Summary statistics: units**					
Others	1.79 (0.73)	1.39 (0.66)	2.33 (1.06)	1.51 (0.93)	1.93 (0.85)
Rheumatology	2.54 (1.04)	2.14 (1.28)	2.82 (1.14)	2.43 (1.31)	2.76 (1.33)
Geriatrics Medicine	2.05 (0.74)	1.48 (0.71)	2.84 (1.01)	1.79 (0.99)	2.02 (0.96)
Infectious Diseases	2.16 (0.78)	1.72 (0.77)	2.76 (1.11)	1.83 (1.12)	2.36 (1.13)
Nephrology	2.28 (0.74)	1.53 (0.51)	3.13 (1.21)	2 (1.22)	2.46 (1.26)
Hematology	2.12 (0.67)	1.46 (0.61)	2.74 (0.81)	1.97 (1.05)	2.31 (0.91)

* Mean (SD).

**Table 3:** Pre- and post-vaccination comparison of individual VAX scale items (N = 90).

Item	Pre (n = 90)	Post (n = 90)	Difference *	95% CI **	*p*-Value ***
**I feel safe after being vaccinated**			2.9	2.6, 3.1	<0.001
Mean (SD)	1.76 (0.94)	4.65 (0.80)			
Median [IQR]	1.00 [1.00, 2.00]	5.00 [5.00, 5.00]			
Missing (N)	0	1			
**I can rely on vaccines to stop serious infectious diseases**			2.9	2.7, 3.2	<0.001
Mean (SD)	1.73 (1.06)	4.69 (0.78)			
Median [IQR]	2.00 [1.00, 2.00]	5.00 [5.00, 5.00]			
Missing (N)	0	1			
**I feel protected after getting vaccinated**			3.0	2.7, 3.2	<0.001
Mean (SD)	1.70 (0.91)	4.70 (0.74)			
Median [IQR]	1.00 [1.00, 2.00]	5.00 [5.00, 5.00]			
Missing (N)	0	1			
**Although most vaccines appear to be safe, there may be problems that we have not yet discovered**			−1.1	−1.5, −0.76	<0.001
Mean (SD)	2.90 (1.23)	1.79 (1.09)			
Median [IQR]	3.00 [2.00, 4.00]	1.00 [1.00, 2.00]			
Missing (N)	1	1			
**Vaccines can cause unforeseen problems in children**			−1.1	−1.4, −0.80	<0.001
Mean (SD)	2.65 (1.25)	1.53 (0.87)			
Median [IQR]	3.00 [2.00, 3.00]	1.00 [1.00, 2.00]			
Missing (N)	2	1			
**I worry about the unknown effects of vaccines in the future**			−0.84	−1.2, −0.53	<0.001
Mean (SD)	2.38 (1.25)	1.55 (0.85)			
Median [IQR]	2.00 [1.00, 3.00]	1.00 [1.00, 2.00]			
Missing (N)	0	1			
**Vaccines make a lot of money for pharmaceutical companies, but do not do much for regular people**			−1.1	−1.4, −0.78	<0.001
Mean (SD)	2.24 (1.38)	1.17 (0.61)			
Median [IQR]	2.00 [1.00, 3.00]	1.00 [1.00, 1.00]			
Missing (N)	0	1			
**Authorities promote vaccination for financial gain, not for people’s health**			−0.81	−1.1, −0.52	<0.001
Mean (SD)	1.94 (1.33)	1.15 (0.61)			
Median [IQR]	1.00 [1.00, 3.00]	1.00 [1.00, 1.00]			
Missing (N)	1	1			
**Vaccination programs are a big con**			−0.52	−0.80, −0.23	<0.001
Mean (SD)	1.64 (1.20)	1.13 (0.57)			
Median [IQR]	1.00 [1.00, 2.00]	1.00 [1.00, 1.00]			
Missing (N)	0	1			
**Natural immunity lasts longer than a vaccination**			−0.85	−1.2, −0.53	<0.001
Mean (SD)	2.49 (1.34)	1.64 (0.68)			
Median [IQR]	3.00 [1.00, 3.00]	2.00 [1.00, 2.00]			
Missing (N)	2	1			
**Natural exposure to viruses and germs gives the safest protection**			−0.43	−0.73, −0.13	0.005
Mean (SD)	2.07 (1.22)	1.64 (0.68)			
Median [IQR]	2.00 [1.00, 3.00]	2.00 [1.00, 2.00]			
Missing (N)	1	1			
**Being exposed to diseases naturally is safer for the immune system than being exposed through vaccination**			−0.51	−0.85, −0.17	0.003
Mean (SD)	2.15 (1.37)	1.64 (0.63)			
Median [IQR]	2.00 [1.00, 3.00]	2.00 [1.00, 2.00]			
Missing (N)	1	1			

* Difference: post—pre. ** CI = Confidence Interval. *** Permutation Paired *t*-test with Welch correction.

**Table 4:** Adverse events and breakthrough infections following herpes zoster vaccination, ordered by frequency.

	Num. Patients	%
**Side Effects: 1st dose**		
No	72	80.00
Fatigue, chills, fever	11	12.22
Pain, redness or swelling at the injection site	5	5.56
General discomfort	5	5.56
Doesn’t know/Can’t remember	2	2.22
Stomach and digestive disorders (including nausea, vomiting, diarrhea, abdominal pain)	1	1.11
Myalgia	1	1.11
Headache	1	1.11
Tingling at the injection site	1	1.11
Lymphadenopathy	0	0
**Side Effects: 2nd dose**		
No	67	74.44
Fatigue, chills, fever	16	17.78
Pain, redness or swelling at the injection site	7	7.78
General discomfort	7	7.78
Stomach and digestive disorders (including nausea, vomiting, diarrhea, abdominal pain)	2	2.22
Tingling at the injection site	2	2.22
Arthralgia	1	1.11
Myalgia	1	1.11
Headache	1	1.11
Arthralgia	0	0
Lymphadenopathy	0	0
Doesn’t know/Can’t remember	0	0
**Breakthrough Infections**		
No	87	96.67
Yes, after 2nd dose—*of which*	3	3.33
Oral	2	66.67 *
Lumbar	1	33.33 *
Ophthalmic	1	33.33 *
Yes, after 1st dose	0	0

* Note: The sum exceeds 100%, as one case involved was both ophthalmic and oral.

## Data Availability

Data supporting the conclusions of the article will be made available by the authors upon reasonable request.

## Supplementary Materials

The following supporting information can be downloaded at: https://www.mdpi.com/article/10.3390/vaccines13080843/s1, Methods S1: Minimization of bias, Timing of the study, Description of the assessment tool; Table S1: Linear regression analysis of factors associated with overall vaccine hesitancy; Table S2: Linear regression analysis of factors associated with mistrust of vaccine benefit; Table S3: Linear regression analysis of factors associated with worries about unforeseen future effects; Table S4: Linear regression analysis of factors associated with concerns about commercial profiteering; Table S5: Linear regression analysis of factors associated with preference for natural immunity.

## Abbreviations

The following abbreviations are used in this manuscript:

HZ	Herpes Zoster
PHN	Postherpetic Neuropathy
VZV	Zostavax
RZV	Recombinant vaccine adjuvanted with the glycoprotein E subunit of VZV Shingrix
PNPV	National Vaccine Prevention Plan
VH	Vaccine Hesitancy
FPG	Fondazione Policlinico Universitario Agostino Gemelli
IRCCS	Istituto di Ricovero e Cura a Carattere Scientifico
A.S.L.	Azienda Sanitaria Locale
HUs	Hospital Units
VAX	Vaccination Attitudes Examination
SD	Standard Deviations
IQR	Interquartile Ranges
