# Supplementary material for: Understanding Attitudes Toward Zoster Vaccination in the Hospital Setting: A Multidisciplinary Model to Contrast Vaccine Hesitancy in Fragile Patients—A Prospective Longitudinal Observational Study
Source: Vaccines (Basel). 2025 Aug 8;13(8):843. doi: 10.3390/vaccines13080843 (PMC12390494; doi:10.3390/vaccines13080843)
Supplement: Supplementary file 1 [file vaccines-13-00843-s001.zip › vaccines-3780094-supplementary.pdf]

Methods S1

Minimization of bias

Selection bias was minimized by including all adult patients referred to the counseling program at Fondazione Policlinico Universitario A. Gemelli (FPG) during the study period. Eligibility criteria were applied consistently and independently of patients' initial vaccine attitudes, ensuring a representative cohort of individuals.

Information bias was minimized by using the validated 12-item Vaccination Attitudes Examination (VAX) scale to assess vaccine hesitancy. Data were collected through structured interviews conducted by trained personnel using standardized forms to reduce variability in administration and misclassification of responses.

Confounding was managed by identifying key variables (e.g., age, sex, comorbidities), based on existing literature and clinical relevance. These were used in stratified analyses and were included in multivariable models.

Timing of the study

The study was divided into three phases.

[T1] The initial phase consisted of the patients' vaccination history reconstruction, characterized by the assessment of vaccination status, indications for further vaccination (adverse events in previous vaccinations), and current or planned therapy for immunosuppressive disorders or/and splenectomy and/or dialysis; in this phase, all patients completed the VAX Scale, a literature-validated instrument for the study of VH.

[T2] In the second phase, patients underwent multidisciplinary vaccination counseling focused on preventing HZ and counteracting VH. The multidisciplinary team comprised: infectious diseases specialist, public health specialist, hematologist, rheumatologist, nephrologist and geriatrician. Team composition varied based on individual patient characteristics, with the public health specialist collaborating with the most appropriate clinical specialist for each case. Then, after signing informed consent, they were vaccinated with 2 doses of RZV, with a timing adapted to the patient's primary pathology, immunosuppressive therapy taken as recorded in their medical records, and the indications of the vaccine datasheet. The interval between doses, according to the vaccine datasheet, is two months, but may be reduced to one month for immunocompromised patients undergoing immunomodulatory therapy or cancer patients awaiting chemotherapy who would benefit from vaccination before receiving treatment. A dedicated multidisciplinary hospital team was selected to carry out RZV vaccination weekly; all the necessary resources for vaccination were made available (workstation, medical bed, stretcher, crash cart, refrigerator, available vaccine products, and materials for vaccination practice such as syringes, disinfectants, cotton, plasters, biological cassettes, emergency trolley). Through collaboration with out-of-hospital health services (ASL Roma 1, local health authority), RZV vaccine was made available at the FPG, in totally free mode. Medical and nursing staff at the FPG hospital vaccine center was responsible for follow-up of vaccinated patients, recording any adverse reactions and/or HZ infections [1].

[T3] Finally, after 365 days had elapsed for each patient since the second dose of vaccine, a patients who had received both doses of vaccine were recalled by phone, aimed at assessing the presentation of side effects after the first or second dose; specifically, each patient was asked whether they had experienced one or more side effects from a predefined list, which had been developed based on the potential adverse events reported in the vaccine datasheet.. In addition, they were asked about the occurrence of breakthrough infections, and, each of them was re-administered the VAX Scale questionnaire to assess any changes in VH. Patients who could not be reached after at least three separate contact attempts on different days were excluded from the follow-up analysis.

Description of the assessment tool

As mentioned before, the assessment of attitudes and intentions toward vaccines was conducted using the Italian adaptation of the 12-item VAX scale, categorized into four distinct subgroups as outlined in

previous research: 1) mistrust of vaccine benefit, 2) worries about unforeseen future effects, 3) concerns about commercial profiteering, and 4) preference for natural immunity. A Likert scale from 1 to 6 is used to score each item, where 1 indicates a "strongly disagree" response and 6 denoting a "strongly agree" response [2]. Among these questions, the scores for the first, second, and third questions are subtracted from the total, while the scores for the other questions are added to the total score. Overall, a higher VAX score indicates a more intense level of anti-vaccine attitude.

The VAX scales can be further classified according to item numbers to which corresponds a different calculation to be applied for the final score: items 1-3 relate to trust in vaccines and should be considered as antithetical to VH, thus subtracted from the score value, while items 4-6 relate to concerns about unanticipated future effects, items 7-9 relate to concerns about commercial gain, and items 10-12 relate to preference for natural immunity and all should be assessed as concordant with the presence of hesitancy thus added to the score value.

Previous research examining vaccination intentions for COVID-19 infection has shown that the subject survey approach has a high degree of reliability [3,4]. A range of >4 out of a maximum of 6 indicated a high level of concern, >2 indicated an intermediate level, and <2 indicated a low level of concern. These scores were indicative of the extent of individuals' negative attitudes toward the vaccine.

The Italian adaptation of the VAX scale demonstrated reliability and discriminant and convergent validity. The scale manifested substantial adherence, thus confirming its ability to identify individuals in the Italian population who manifest hesitation toward the vaccine [5].

Bibliography

1. Nota regionale prot. n.927983 del 12 novembre 2021. Vaccino ricombinante adiuvato Shingrix. Ripartizione tra le Aziende USL dell'approvvigionamento annuale di prodotto ed ulteriori indicazioni per il suo utilizzo. Available from: from https://www.regione.lazio.it. [Accessed 2024 Nov 16].
2. Martin LR, Petrie KJ. Understanding the Dimensions of Anti-Vaccination Attitudes: the Vaccination Attitudes Examination (VAX) Scale. Ann Behav Med. 2017 Oct;51(5):652–60.
3. Cummings PE, Lakoh S, Yendewa SA, Massaquoi SPE, James PB, Sahr F, et al. Understanding COVID-19 Vaccine Uptake and Hesitancy among People with HIV in Freetown, Sierra Leone: A Cross-Sectional Study. Vaccines (Basel). 2023 Nov 2;11(11):1685.
4. Huynh HP. Examining four types of anti-vaccination attitudes prior to and during the COVID-19 pandemic. Curr Psychol. 2022 Nov 10;1–8.
5. Bruno F, Laganà V, Pistininzi R, Tarantino F, Martin L, Servidio R. Validation and psychometric properties of the Italian Vaccination Attitudes Examination (VAX-I) scale. Curr Psychol. 2022 May 27;1–11.

Table S1. Linear regression analysis of factors associated with overall vaccine hesitancy.

Variable	Coefficient	Std. Error	t-value	p-value	[95% Conf.	Interval]	Significance
Female	.174	.181	0.96	.337	-.184	.532	
Age	.028	.002	12.44	0	.024	.033	***
Other	-.016	.353	-0.05	.964	-.713	.681	
Rheumatology	1.121	.21	5.35	0	.708	1.535	***
Geriatrics	.222	.241	0.92	.359	-.255	.699	
Nephrology	.24	.505	0.47	.636	-.758	1.238	
Hematology	.073	.328	0.22	.823	-.574	.721	

Base categories: Gender - Male; Division - Infectious Diseases

Mean dependent var	2.291	SD dependent var	0.936
R-squared	0.822	Number of obs	167
F-test	105.531	Prob > F	0.000
Akaike crit. (AIC)	502.163	Bayesian crit. (BIC)	523.988

**** p<.01, ** p<.05, * p<.1*

Table S2. Linear regression analysis of factors associated with mistrust of vaccine benefit.

Variable	Coefficient	Std. Error	t-value	p-value	[95% Conf.	Interval]	Significance
Female	.106	.191	0.55	.581	-.272	.484	
Age	.022	.002	9.10	0	.017	.026	***
Other	-.004	.372	-0.01	.992	-.739	.731	
Rheumatology	1.31	.221	5.92	0	.873	1.746	***
Geriatrics	.078	.255	0.30	.761	-.425	.581	
Nephrology	-.015	.533	-0.03	.978	-1.067	1.038	
Hematology	-.099	.346	-0.29	.775	-.782	.584	

Base categories: Gender - Male; Division - Infectious Diseases

Mean dependent var	1.852	SD dependent var	1.087
R-squared	0.737	Number of obs	167
F-test	64.004	Prob > F	0.000
Akaike crit. (AIC)	520.022	Bayesian crit. (BIC)	541.848

**** p<.01, ** p<.05, * p<.1*

Table S3. Linear regression analysis of factors associated with worries about unforeseen future effects.

Variable	Coefficient	Std. Error	t-value	p-value	[95% Conf.	Interval]	Significance
Female	.417	.214	1.95	.053	-.006	.84	*
Age	.035	.003	13.27	0	.03	.041	***
Other	.043	.413	0.11	.916	-.771	.858	
Rheumatology	.549	.244	2.25	.026	.066	1.031	**
Geriatrics	.382	.289	1.32	.188	-.188	.952	
Nephrology	.354	.589	0.60	.549	-.81	1.517	
Hematology	.018	.382	0.05	.962	-.737	.773	

Base categories: Gender - Male; Division - Infectious Diseases

Mean dependent var	2.769	SD dependent var	1.056
R-squared	0.831	Number of obs	166
F-test	111.987	Prob > F	0.000
Akaike crit. (AIC)	550.148	Bayesian crit. (BIC)	571.931

**** p<.01, ** p<.05, * p<.1*

Table S4. Linear regression analysis of factors associated with concerns about commercial profiteering.

Variable	Coefficient	Std. Error	t-value	p-value	[95% Conf.	Interval]	Significance
Female	.006	.223	0.03	.979	-.435	.447	
Age	.025	.003	9.05	0	.02	.031	***
Other	-.107	.434	-0.25	.806	-.965	.751	
Rheumatology	1.454	.258	5.64	0	.944	1.963	***
Geriatrics	.211	.297	0.71	.48	-.376	.798	
Nephrology	.32	.622	0.51	.607	-.908	1.548	
Hematology	.244	.404	0.60	.547	-.554	1.041	

Base categories: Gender - Male; Division - Infectious Diseases

Mean dependent var	2.095	SD dependent var	1.287
R-squared	0.727	Number of obs	167
F-test	60.717	Prob > F	0.000
Akaike crit. (AIC)	571.584	Bayesian crit. (BIC)	593.410

*** p<.01, ** p<.05, * p<.1

Table S5. Linear regression analysis of factors associated with preference for natural immunity.

Variable	Coefficient	Std. Error	t-value	p-value	[95% Conf.	Interval]	Significance
Female	.1	.229	0.43	.665	-.353	.552	
Age	.031	.003	10.84	0	.025	.036	***
Other	-.05	.444	-0.11	.911	-.926	.826	
Rheumatology	1.186	.263	4.51	0	.666	1.706	***
Geriatrics	.074	.308	0.24	.81	-.535	.684	
Nephrology	.315	.635	0.50	.621	-.939	1.568	
Hematology	.122	.412	0.30	.768	-.692	.935	

Base categories: Gender - Male; Division - Infectious Diseases

Mean dependent var	2.450	SD dependent var	1.180
R-squared	0.767	Number of obs	166
F-test	74.944	Prob > F	0.000
Akaike crit. (AIC)	574.901	Bayesian crit. (BIC)	596.685

*** p<.01, ** p<.05, * p<.1
